# Optimizing Network Connectivity for Mobile Health Technologies in sub-Saharan Africa

**DOI:** 10.1371/journal.pone.0045643

**Published:** 2012-09-28

**Authors:** Mark J. Siedner, Alexander Lankowski, Derrick Musinga, Jonathon Jackson, Conrad Muzoora, Peter W. Hunt, Jeffrey N. Martin, David R. Bangsberg, Jessica E. Haberer

**Affiliations:** 1 Massachusetts General Hospital, Harvard Medical School, Boston, Massachusetts, United States of America; 2 Mbarara University of Science and Technology, Mbarara, Uganda; 3 Dimagi, Inc., Boston, Massachusetts, United States of America; 4 University of California San Francisco, San Francisco, California, United States of America; 5 Ragon Institute of Massachusetts General Hospital, Massachusetts Institute of Technology, and Harvard Medical School, Boston, Massachusetts, United States of America; Tulane University, United States of America

## Abstract

**Background:**

Mobile health (mHealth) technologies hold incredible promise to improve healthcare delivery in resource-limited settings. Network reliability across large catchment areas can be a major challenge. We performed an analysis of network failure frequency as part of a study of real-time adherence monitoring in rural Uganda. We hypothesized that the addition of short messaging service (SMS+GPRS) to the standard cellular network modality (GPRS) would reduce network disruptions and improve transmission of data.

**Methods:**

Participants were enrolled in a study of real-time adherence monitoring in southwest Uganda. In June 2011, we began using Wisepill devices that transmit data each time the pill bottle is opened. We defined network failures as medication interruptions of >48 hours duration that were transmitted when network connectivity was re-established. During the course of the study, we upgraded devices from GPRS to GPRS+SMS compatibility. We compared network failure rates between GPRS and GPRS+SMS periods and created geospatial maps to graphically demonstrate patterns of connectivity.

**Results:**

One hundred fifty-seven participants met inclusion criteria of seven days of SMS and seven days of SMS+GPRS observation time. Seventy-three percent were female, median age was 40 years (IQR 33–46), 39% reported >1-hour travel time to clinic and 17% had home electricity. One hundred one had GPS coordinates recorded and were included in the geospatial maps. The median number of network failures per person-month for the GPRS and GPRS+SMS modalities were 1.5 (IQR 1.0–2.2) and 0.3 (IQR 0–0.9) respectively, (mean difference 1.2, 95%CI 1.0–1.3, p-value<0.0001). Improvements in network connectivity were notable throughout the region. Study costs increased by approximately $1USD per person-month.

**Conclusions:**

Addition of SMS to standard GPRS cellular network connectivity can significantly reduce network connection failures for mobile health applications in remote areas. Projects depending on mobile health data in resource-limited settings should consider this upgrade to optimize mHealth applications.

## Introduction

Cellular phone access in sub-Saharan Africa increased from approximately 5 to 70% during 2000 to 2008, to reach over 380 million users [1], [2]. The widespread ownership of cellular phones and corresponding wireless network infrastructure enable public health programs to leverage mobile health (mHealth) technologies, with a goal of improving health care monitoring and delivery in resource-limited settings via real-time communication across large catchment areas. Consequently, there has been an international effort to coordinate the design, testing, and implementation of novel health devices in low and middle-income countries [3], [4]. Early successes from these technologies have included drug adherence monitoring [5], [6], strengthening of patient provider communication [7], [8], and improved healthcare quality control [9].

Notwithstanding some successful examples, few successful mHealth interventions have been described in the developing world [10], [11]. A defining feature of mobile applications is reliance on cellular phone networks for transfer of information. Fluctuations in network availability in rural areas is a frequently reported challenge [12], and especially limits the functionality of real-time monitoring systems [13], [14].

In June 2011, we began performing real-time anti-retroviral therapy (ARV) adherence monitoring as part of a cohort study of HIV-infected patients in rural Uganda using pill bottles that transmit messages via local cellular phone networks. These devices initially employed standard general packet radio service (GPRS) cell phone technology [15]. After experiencing high rates of network disruptions and failed data transmissions, we upgraded our devices to include both GPRS and short message services (GPRS+SMS), which allows a backup system of data transmission when the GPRS connection fails. We describe here our experience with both services including difference in network failure frequency and a geospatial depiction of these differences to inform programs using real-time mHealth interventions in resource-limited settings.

**Table 1:** Demographic characteristics for participants (n = 155) with wireless adherence monitors in southwestern Uganda.

Characteristic	Summary Statistic
Age (median, IQR)	40 (33–46)
Female (%)	73
Altitude in meters (median, IQR) (n = 106)	1.420 (1401–1439)
Distance from closest water source <500 meters (%)	71
Access to home electricity	17
Travel time to clinic <60 minutes (%)	39

## Materials and Methods

### Study Design

The study was approved by the ethics review committees of the Mbarara University of Science and Technology, the Uganda National Council of Science and Technology, and Partners Healthcare and was registered with Clinicaltrials.gov (NCT01596322). All participants gave written informed consent. Study participants were enrolled in the Uganda AIDS Rural Treatment Outcomes Study (UARTO), a prospective cohort study that began in 2005, designed to measure correlates of HIV-adherence in rural Uganda [16]. Participants were enrolled from the Immune Suppression Syndrome Clinic in Mbarara, Uganda at the time of ART initiation. We collected socio-demographic characteristics at baseline and completed a home visit to record global positioning systems (GPS) coordinates. In June 2011 we began using Wisepill devices (Wisepill, Somerset, South Africa), which are pill containers that contain subscriber identity module (SIM) cards. The devices transmit a standard GPRS cell phone message via the cellular phone network to a server hosted in South Africa each time the pill bottle is opened [5]. We began systematic recording of transmission interruptions in August 2011, which marks the start of our study analysis time. Any lapse in signal >48 hours can represent either a treatment interruption or a network transmission failure. In cases of network interruptions, data was “back-filled” to the server when the network signal is re-established. In cases where no data was received by the Monday of the following week, study staff visited participant homes to conduct a structured interview and assess for network signal strength to determine if the missing data represented medication non-adherence or network connection failures. A network failure was thus defined as any lapse in pill bottle openings for >48 hours that was later back-filled to the server before or during a home visit. Due to high rates of network failures using GPRS messages, we began upgrading the devices to enable both GPRS and SMS data transmission in September 2011. In contrast to the GPRS messages, SMS messages are sent to a sever in Uganda via a longcode phone number hosted by a third-party content aggregator (Yo! Technologies). Upon receipt of the SMS message, the content aggregator sends a message to the server in South Africa via the short message peer-to-peer modality.

**Table 2:** Rates of wireless network connectivity failures for wireless adherence devices by general packet radio service (GPRS) versus short message service (GPRS+SMS) compatibility in 155 study participants in southwestern Uganda.

	GPRS	GPRS+SMS
Total Study Time (days)	16,445	11,331
Total backfills (n)	948	253
Participant study time in days (median, IQR)	104 (82–120)	72 (58–93)
Number of network failures per 28 days (median, IQR)*	1.5 (1.0–2.2)	0.3 (0–0.9)
No network failures (n, %)	5 (3%)	72 (46%)
<1 failures/28 days (n, %)	37(24%)	53 (34%)
>1 failures/28 days (n, %)	115 (73%)	32 (20%)
Median network failure rate per 28 days (IQR)*	1.5 (1.0–2.1)	0.3 (0–0.9)

* p-value for difference in mean network failures by Wilcoxon signed-rank test = <0.0001.

GPRS = general packet radio service compatible.

GPRS+SMS = general packet radio service and short message service compatible.

### Statistical Methods

UARTO participants were included in network connectivity analysis if they had received a Wisepill device and had a minimum of seven days of GPRS and seven days of GPRS+SMS observation time. We limited the GPS map to participants who had GPS coordinates taken since their last change in home residence. We summarized socio-demographic characteristics for participants and compared network failure rates during GPRS and GPRS+SMS periods using Wilcoxon signed-rank testing. We also summarized failure rates by calendar time to evaluate whether temporal changes in cellular network reliability could explain differences in connectivity rates. We created geospatial maps to graphically depict changes in failure frequency by geographic location for participants who had GPS coordinates completed since their last change in residence. We created two maps: one each for the GPRS and GPRS+SMS study periods. We entered participant home GPS coordinates into an online GPS mapping software (GPSVisualizer.com) along with their categorized, color-coded frequency of network failure frequency (green = 0 failures, yellow = 0–1 failures/28 days, red = >1 failure/28 days) to produce topographical maps with Google Maps.

## Results

One hundred and fifty-seven participants were eligible for the network failure analysis. Participants had a median of 40 years of age and were 73% female (Table 1). The study population was largely rural as evidenced by the fact that 39% reported at least one hour of transport time to arrive at the clinic, 17% reported access to home electricity and 29% stated they live at least 500 meters from the closest water source.

Study participants had a median of 104 days of GPRS study time (IQR 82–120 days) and 72 days of GPRS+SMS study time (IQR 58–93 days). We recorded a total of 948 network failures in 16,445 GPRS study days and 253 network failures in 11,331 GPRS+SMS study days. Participants had a median of 1.5 failures/28 days during GPRS time and a median of 0.3 failures/28days during GPRS+SMS time, for a mean difference of 1.2 more failures per person-month during GPRS than GPRS+SMS periods (95% CI 1.0–1.3, t = 15.0, p<0.0001), (Table 2).

**Table 3:** Change in network failure rate category from before to after upgrade to short messaging service.

	GPRS Only			
GPRS+SMS Compatible	0 Failures per Month	0–1 Failuresper Month	>1 Failureper Month	Total
0 Failures/Month	4	**25**	**43**	72
0–1 Failures/Month	***1***	10	**42**	53
>1 Failure/Month	***0***	***2***	30	32
	5	37	115	157

Bolded text boxes represent participants who had improved wireless connectivity with the GPRS+SMS upgrade; bolded and italicized text boxes represent participants who worse connectivity with the upgrade.

**Figure 1:**
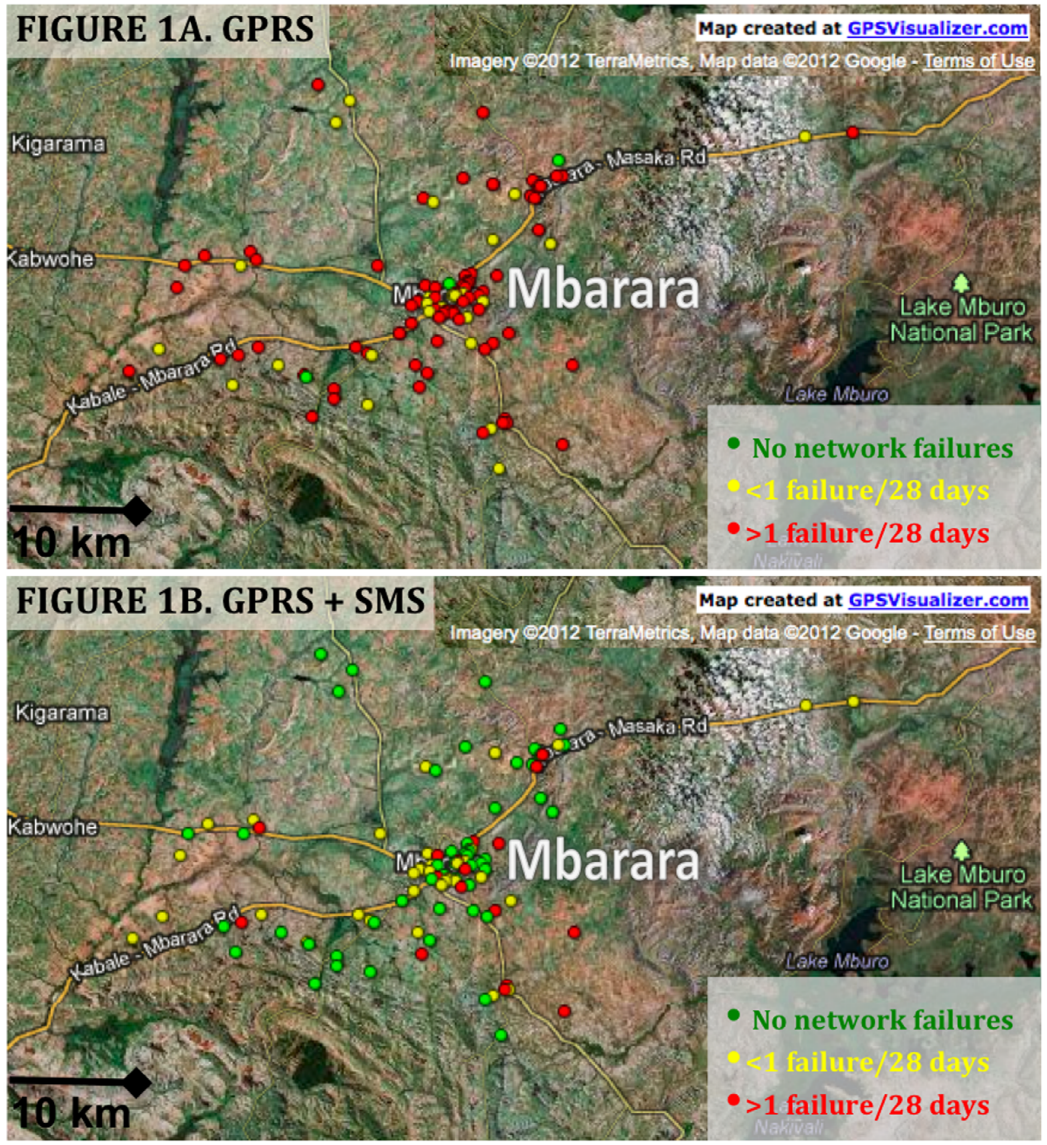
Geospatial map of participant homes (n = 100) in a study of real-time adherence monitoring using devices that transmit bottle openings via cellular phone networks. Panel A shows connectivity failure rates for devices with general packet radio service (GPRS) network compatibility only. Panel B shows connectivity failure rates for devices with general packet radio service and short message service (GPRS+SMS) compatibilities. Green markers represent households with no history of network failures; yellow markers for households with 0–1 failures/28 days; red markers for households with >1 failure/28 days.

One hundred ten participants (70%) had improved connectivity with the upgrade, 44 (28%) had little change in network failure rates and 3 (2%) had increased network interruptions (Table 3). One hundred one participants had GPS coordinates since their last move and were included in the geospatial maps (Figure 1). Connectivity was improved with the SMS upgrade both in urban and remote areas with varied topography throughout the region. The median failure rate for GPRS+SMS was significantly lower than for GPRS for four of the six study months. (August 2011∶0 vs. 1.1, September 2011∶0 vs. 0.9, October 2011∶0. Vs 0.9; November 2011∶0 vs. 0.9 failures/28 days). The rates were similar due to relatively high rates of GPRS+SMS failures in December 2011 (0.9 vs. 0.9 failures/28 days) and relatively low rates of GPRS failures in January 2012 respectively (0 vs. 0 failures/28 days).

Network connection costs in Uganda for Wisepill devices with GPRS functionality include approximately $USD1.25 for the SIM card and an additional $USD0.80 for three months of airtime to transmit data. We pay study costs of $USD130.00 per month for a contract with a Ugandan SMS Aggregator to receive SMS messages. It costs approximately $USD0.03 for each message sent by the devices per day, or an estimated $USD1.00 per participant per month if an SMS message is sent daily.

## Discussion

We experienced an 80% decrease in network connection failures with our real-time wireless medication adherence monitors after upgrading from the standard GPRS cell phone network data transmission connection to an SMS-enabled system. Improvements in connectivity were seen throughout southwestern Uganda and cost approximately $1USD per study participant per month. Health programs developing mobile health applications that rely on cell phone network connectivity in rural settings should consider this upgrade to improve delivery of data and health communications.

mHealth applications hold incredible promise to improve health care delivery in the developing world [4]. However there are several barriers to successful implementation of these applications including behavioral [17], low end-user literacy [18], and technical [19] issues. Realizing the anticipated potential of mHealth interventions will require a multi-factorial evaluation [20]. All applications rely on reliable, often real-time data transmission, and often from remote regions where wireless connectivity might be poor [12], [13]. In these settings, the causes of such failures can be related to: 1) fluctuations in wireless signals due to weather, power and other cell phone tower infrastructure issues; 2) limited compatibility of mHealth applications to transmit data (lack of GPRS or SMS); and/or 3) complete lack of wireless infrastructure. Our findings suggest SMS back-up data transmission can decrease the frequency of network connection failures.

Our findings are generalizable to groups working in similar settings with low or inconsistent penetration of cellular phone network signals. In contrast, areas with strong cellular connectivity are less likely to benefit from the addition of SMS transmission capabilities. Moreover, applications that primarily rely on SMS messaging to send data already benefit from the improvements we describe here. However, most applications that utilize standard cell phone connectivity, including real-time monitoring devices, biosensors, and diagnostic applications depend on GPRS technology for data transmission and thus might require software alterations to permit SMS communication. Though SMS messages are less susceptible to variations of network connectivity, they are also susceptible to failure related to both telecommunication company transmission failures, and third-party transmission failures. Our findings all most generalizable to groups working with third-party aggregators, and might be improved further for those who work directly with telecommunications companies to host message processing. Thus, groups considering this upgrade for mHealth applications should also consider options for and connectivity implications related to hosting a phone number and computer server (*i.e.* SMS gateway), to receive and store data from messages.

### Conclusions

In summary, addition of SMS functionality to real-time mHealth applications that rely on GPRS networks in resource-limited settings might substantially improve data transmission rates. The costs of this upgrade are relatively low, though could be an added to challenge to publicly funded programs. Further evaluation and dissemination of information about network connectivity challenges for mobile health applications will improve the applicability of mHealth interventions.
